# Effectiveness of an insurance enrollment support tool on insurance rates and cancer prevention in community health centers: a quasi-experimental study

**DOI:** 10.1186/s12913-021-07195-5

**Published:** 2021-10-30

**Authors:** Huguet Nathalie, Valenzuela Steele, Marino Miguel, Moreno Laura, Hatch Brigit, Baron Andrea, J. Cohen Deborah, E. DeVoe Jennifer

**Affiliations:** 1 Department of Family Medicine, Oregon Health & Science University, 3181 SW Sam Jackson Park Rd, Portland, OR 97239 USA; 2 Division of Biostatistics, School of Public Health, Oregon Health & Science University – Portland State University, 3181 SW Sam Jackson Park Rd, Portland, OR 97239 USA; 3 Research Department, OCHIN Inc, 1881 SW Naito Pkwy, Portland, OR 97201 USA

**Keywords:** Health insurance, Health information technology, Electronic health record tool, Implementation science, Navigator, Medicaid

## Abstract

**Background:**

Following the ACA, millions of people gained Medicaid insurance. Most electronic health record (EHR) tools to date provide clinical-decision support and tracking of clinical biomarkers, we developed an EHR tool to support community health center (CHC) staff in assisting patients with health insurance enrollment documents and tracking insurance application steps. The objective of this study was to test the effectiveness of the health insurance support tool in (1) assisting uninsured patients gaining insurance coverage, (2) ensuring insurance continuity for patients with Medicaid insurance (preventing coverage gaps between visits); and (3) improving receipt of cancer preventive care.

**Methods:**

In this quasi-experimental study, twenty-three clinics received the intervention (EHR-based insurance support tool) and were matched to 23 comparison clinics. CHCs were recruited from the OCHIN network. EHR data were linked to Medicaid enrollment data. The primary outcomes were rates of uninsured and Medicaid visits. The secondary outcomes were receipt of recommended breast, cervical, and colorectal cancer screenings. A comparative interrupted time-series using Poisson generalized estimated equation (GEE) modeling was performed to evaluate the effectiveness of the EHR-based tool on the primary and secondary outcomes.

**Results:**

Immediately following implementation of the enrollment tool, the uninsured visit rate decreased by 21.0% (Adjusted Rate Ratio [RR] = 0.790, 95% CI = 0.621–1.005, *p* = .055) while Medicaid-insured visits increased by 4.5% (ARR = 1.045, 95% CI = 1.013–1.079) in the intervention group relative to comparison group. Cervical cancer preventive ratio increased 5.0% (ARR = 1.050, 95% CI = 1.009–1.093) immediately following implementation of the enrollment tool in the intervention group relative to comparison group. Among patients with a tool use, 81% were enrolled in Medicaid 12 months after tool use. For the 19% who were never enrolled in Medicaid following tool use, most were uninsured (44%) at the time of tool use.

**Conclusions:**

A health insurance support tool embedded within the EHR can effectively support clinic staff in assisting patients in maintaining their Medicaid coverage. Such tools may also have an indirect impact on evidence-based practice interventions, such as cancer screening.

**Trial registration:**

This study was retrospectively registered on February 4th, 2015 with Clinicaltrials.gov (#NCT02355262). The registry record can be found at https://www.clinicaltrials.gov/ct2/show/NCT02355262.

## Background

Lack of health insurance in the United States is linked to unmet healthcare needs [1], intermittent access to necessary treatment and services (e.g., medication) [2–7], and lower likelihood of receipt of evidence-based services as recommended [8–15], compared to those with insurance coverage. These delays can lead to higher rates of disease incidence and mortality, and increased healthcare costs [11, 16–24]. In contrast, greater access to health insurance is associated with lower mortality rates [25], lower out-of-pocket expenditures and medical debts (especially with public insurance), greater self-reported mental and physical health [26]. The positive impact of health insurance on life expectancy is due, in part, to health insurance facilitating receipt of recommended care, especially preventive care services essential for early detection and management of cancer and other chronic diseases. For instance, timely receipt of cancer screening and prevention has contributed to the increase in cancer survival rates [27].

Based on evidence linking health insurance to better health outcomes, the Patient Protection and Affordable Care Act (ACA) was passed in 2010 to expand access to health insurance coverage in the United States (US) [28–30], and between 2013 and 2018 the number of uninsured people in the US dropped from 44.8 million to 27.9 million [1]. As predicted, the ACA expansions in access to health insurance were associated with higher rates of receipt of recommended care, including provision of preventive services [31].

Successfully enrolling in an insurance program and staying enrolled, however, can be difficult and complex, especially for patients eligible for Medicaid insurance. There are numerous barriers [32–34] to both initial and re-enrollment into Medicaid including patient stigma, lack of knowledge on how to apply, a complex application process, and misunderstandings about whether an individual meets all of the eligibility criteria. The ACA led to improvement in re-enrollment with states using available data on the beneficiary to determine ongoing eligibility [35]. That said, if data are incomplete, the beneficiary must fill-out the missing information within a 30-day of the termination period, which can be challenging and if enrollment is not renewed within 90 days of the termination date, the beneficiaries must complete a new application [35]. To help individuals in overcoming these challenges, the Navigator program was implemented as part of the ACA legislation to provide outreach, education, and enrollment assistance to adults seeking or needing health insurance [36]. The Centers for Medicare and Medicaid Services (CMS) provided funds for the navigators who mainly assisted adults with obtaining coverage through health insurance marketplace for the first few years after the ACA. Since then, funds for this navigator program have been reduced substantially [37]. In addition to initial investments in the Navigator program, the Health Resources and Services Administration (HRSA) provided outreach and enrollment assistance grants to federally qualified health centers to assist their patients with obtaining insurance [37]. Over 1000 community health centers (CHCs) have been awarded these HRSA grants [38]. CHCs provide services to nearly 28 million patients every year. The patient population of CHCs is predominately low-income (91% at near or in poverty), racial and ethnic minorities (36% Hispanic/Latino; 22% black), and Medicaid beneficiaries (48%) or uninsured patients (23%). CHCs reduce barriers to cost (through sliding scale fee structures), accept patients without insurance, and tailor services to specific populations (e.g., homeless, non-English speakers) [36].

Unlike the CMS navigator program which is on the decline, the HRSA-funded outreach and enrollment (O&E) efforts supported ongoing work done by eligibility specialists who are located within or directly linked to the CHCs. These O&E specialists assist patients with enrolling and re-enrolling in health insurance, especially with Medicaid. Patients needing assistance may self-identify or be referred at the time of an appointment, or identified for outreach prior to an appointment so that necessary insurance is in place by the time of their appointment. The HRSA O&E awards required CHC grant recipients to track the number of times they assisted individuals with health insurance. Initially, these requirements were complex and included keeping track of the count of persons assisted, applications submitted, and estimation of the number of individuals enrolled.

Most O&E specialist did not have tools embedded within the EHR or other clinical practice management systems to facilitate their O&E work. Yet, recent advances in health information technology (HIT) capabilities could provide opportunities for developing HIT tools to facilitate more timely and effective support for health insurance enrollment and maintenance. Within a hybrid effectiveness-implementation design [39], we designed and implemented a *health insurance support tool* integrated within the electronic health record (EHR) to facilitate the essential services O&E navigators made. By facilitating the services provided by O&E navigators, more patients could be assisted, gain insurance, or avoid insurance gaps improving access to care for these patients. In this two-arm cluster-randomized trial (described elsewhere) [40], we recruited a total of 23 clinics in seven health center systems randomly assigned to one of two intervention arms. Both arms received the intervention (insurance support tool) with the only difference being varying degree of implementation support which lasted 18 months. Qualitative data collection included ethnographic observation and semi-structured interviews with key stakeholders at CHCs focused on barriers and facilitators to adoption of the enrollment tool were also conducted. Details about the implementation of the tool and differences in tool adoption between the two intervention arms appear elsewhere [40–42]. To summarize the findings of the implementation trial: we found that both arms used the tool though adoption was low (16%), but uptake was higher in Arm 2 (received practice coaching). Qualitative interviews identified the importance of perceived relative advantage of the tool, implementation climate, and leadership engagement to as facilitators for adoption [41].

The aim of this quasi-experimental study was to determine whether the use of the EHR insurance support tool assisted with insurance application and insurance gaps, and whether it was associated with improvement in cancer preventive care delivery. Here, we assess whether the tool was effective in improving outcomes considering CHCs, albeit not all, did adopt the tool.

## Methods

### Setting

We recruited CHCs from the OCHIN practice-based research network (PBRN) [43]. OCHIN is a large network of CHCs using a single instance of the Epic EHR. Its centrally hosted Epic EHR is deployed in nearly 100 organizations caring for nearly 2,000,000 patients across 18 states. Participating CHCs were recruited from a subset of OCHIN PBRN members meeting the following criteria: location in a state that expanded Medicaid in 2014, implementation of the OCHIN EHR prior to 2013, and > 1000 adult patients with ≥1 visit in the year prior to the study.

### Intervention: insurance support tool

The insurance support tool (referred to as the “enrollment tool”), described elsewhere [40, 41], consisted of an electronic ‘form’ which appeared alongside typical patient registration processes within the EHR. O&E specialists or other staff who assist patients with registration and insurance enrollment enter the data in the form and use it to track enrollment. The enrollment tool includes the following functionalities:

#### Tracking and documenting

Fillable fields to collect and document insurance enrollment information such as status of insurance application, insurance ID, effective date, eligibility status, number and type of assists provided, total number of individuals assisted, notes, etc.

#### Panel management function

Allows users to (1) run a report of patients with upcoming appointments within 30 days and identify those without health insurance; and (2) run a daily report of health insurance application assistance in progress.

#### Retrospective data report

Reports the number of total individuals assisted with each opened form to generate HRSA quarterly reporting of outreach and enrollment assistance provided.

Tool adoption was measured as the first instance the enrollment form was touched for a specific patient. Though the form could be updated, to assess effectiveness, we utilized the first instance of tool use.

### Comparison group

We used propensity score matching [44] to identify the comparison group of 23 CHCs that most closely resembled the intervention group on clinic and patient characteristics that have the potential to confound the enrollment tool’s effect on health insurance coverage and cancer screening rates. Eligible clinics for the study’s comparison group were matched based on the state of the organization (i.e.*,* comparison clinics had to be in the same states as those in intervention clinics), the total number of patients in 2014 (i.e.*,* prior to intervention initiation), and the percentage of uninsured visits, female gender, and Medicaid beneficiaries. The 23 comparison clinics were selected on a 1:1 ratio based on the nearest available match from the intervention clinics. The intent was to achieve the optimal overall balance in the matching characteristics between the intervention and comparison groups. Balance diagnostics were performed to assess whether the propensity score model had been properly specified [45]. All intervention and matched comparison clinics received HRSA grant funding for outreach and enrollment and are located in Oregon, California, and Ohio.

### Study period

The enrollment tool was implemented mid-September 2016. There are overlapping yet separate study periods for (1) the intent-to-treat analysis assessing tool effectiveness on the primary outcomes of insurance status and cancer screenings at the clinic-level and (2) the effect of the treatment on the treated to evaluate, at the patient-level, the impact among those for whom the tool was used on. For the intent-to-treat analysis, we collected data on tool adoption for 18 months before tool implementation and 18 months after tool implementation. For the analysis estimating the effect of treatment on the treated, the study period started 6 months prior to tool implementation, in order to establish baseline insurance, followed by 18 months of tool implementation, plus an additional 6 months, to allow post-tool follow-up visits and Medicaid application processing time, for a total of 24 months of evaluation.

### Patient inclusion

Patients aged 19–64 with ≥1 ambulatory visit prior to tool use (assessment of insurance status/cancer screenings) were included. EHR data from patients in Oregon CHCs were linked to Medicaid enrollment data from the Oregon Health Authority to determine enrollment status.

### Measures

#### Insurance status

EHR data contain information on payor types as well as billable codes for services performed at each ambulatory care visit; as these data are used for billing purposes, they represent reliable information on insurance status and services received at each visit [46]. For the primary outcome of insurance status, we considered separate monthly insurance rates (including Medicaid, private, other public, and uninsured). Each monthly insurance rate was estimated as the number of monthly encounters paid by a specific insurance type (or self-pay) divided by total visits for patients aged 19–64 years old. For the effect of the treatment on the treated analyses, we used the visit date nearest to the tool use to determine baseline insurance status. Patients could be uninsured or insured by Medicaid, or classified as “other” insurance, which consisted mainly of private insurance and also Medicare and public programs (e.g.*,* grants).

#### Cancer screenings

EHR data were used to assess whether patients were up-to-date with cervical, colorectal, and breast cancer screenings. Services due and services received were identified through procedure codes, diagnosis codes, lab/imaging/scanned results, active problem lists, and longitudinal “health maintenance” records. Services that were ordered but not verified as received (via a direct result or health maintenance result) were not counted. For each measure, individuals were identified as “due” for screening based on sex and age eligibility. For individual measures, patients for whom the screening was not indicated based on special circumstances were excluded (e.g.*,* women with a history of total hysterectomy were excluded from cervical cancer screening). A preventive ratio was calculated for each cancer screening [47, 48]. A preventive ratio is the total person-time covered (after delivery of a particular preventive service) divided by the total person-time eligible for a particular service. This calculation results in a percentage of time “covered” by a preventive service (e.g.*,* a mammogram “covers” an individual for breast cancer screening for 2 years). The percentage ranges from 0 to 100%, where 100% represents complete coverage *(*i.e.*,* services received without delay). Patient-level preventive ratios were averaged across patients within clinics to produce clinic-level measure of performance. We use preventive ratios over conventional metrics (i.e.*,* binary receipt of a service over a certain period) because it provides an estimate of the average time covered (or delayed) by the preventive service as a percentage rather than a dichotomous value. Using a clinic-level preventive ratio for each cancer screening, we are able to assess whether intervention clinics had fewer patients overdue for screenings than comparison clinics.

### Statistical analyses

Unadjusted descriptive statistics of patient characteristics and insurance status were reported at the clinic-level and stratified by intervention group. Characteristics included the average percent of female patients over the entire study period, percent of patients at < 138% of the federal poverty line (FPL), percent speaking English or Spanish, and percent classified as Non-Hispanic White or non-Hispanic minorities (all other races)/Hispanic, and state in which the clinics were located. Chi-square tests and t-tests assessed the differences in characteristics between comparison and intervention groups.

To assess the effectiveness of the enrollment tool on insurance status, we first compared changes in clinic-level rates of insurance pre- versus post-tool implementation in intervention relative to comparison clinics using an intent-to-treat approach. We utilized a comparative interrupted time-series framework to estimate the monthly rate of three types of insurance statuses (uninsured, Medicaid, and private/other insurance) between 23 intervention clinics and 23 comparison clinics, across the 36-month study period. The “interruption”, or the implementation of the enrollment tool, occurred in the middle of the study, where we modeled the possibility of a level change in the monthly rate (i.e.*,* an immediate intervention effect following implementation) and a rate change in the monthly rate (i.e.*,* post-intervention change in the rate over time). Although several modeling options are possible including a difference-in-differences approach, we utilized a comparative interrupted time-series approach as it provides a more flexible inferential framework than difference-in-differences designs given our monthly data collection and study design. Additionally, comparative interrupted time-series has often been noted as a more powerful approach than difference-in-differences [49]. To model the clinic-level monthly insurance rate, we fit a Poisson generalized estimated equation (GEE) model, assuming an autoregressive covariance matrix of degree 1 (AR1) to account for the autocorrelation of monthly observations within clinics. We used the same modeling approach to assess the effectiveness of the enrollment tool on three different cancer screenings: breast cancer, cervical cancer, and colorectal cancer. Overall, our regression models followed the following form:

1 $$ \log \left({Y}_{jt}\right)={B}_0+{B}_1\;{time}_t+{B}_2{post}_t+{B}_3{group}_j+{B}_4{time}_t{group}_j+{B}_5{post}_t{group}_j+{B}_6 Female+{B}_7 FPL138+{B}_8 English+{B}_9 NHW+{B}_{10} State+\log \left({\mu}_{jt}\right) $$

where Y_jt_ is the number of patients in practice *j* who had an insurance visit or cancer screening during time *t*. The trend of the control group is denoted by B_1_. The intervention period is denoted by B_2_, with an indicator of 1 for post_t_ in the last half of the study (18 months) and 0 otherwise. Intercept differences between clinics that were in the intervention group are represented by group_j_. The difference of the linear trend of the intervention group relative to the comparison group in the pre-period is B_4_. B_5_ represents the level change in slope of the treatment on the intervention group in the post-period. B_6_ to B_10_ represent clinic- and state-level characteristics. Lastly, *μ*_*jt*_ is the number of patients in clinic *j* at time *t* who were eligible (i.e., the denominator), for an insurance visit or cancer screening, and the logarithm of this term is considered the offset of a Poisson regression model. To assess the validity of utilizing a comparative interrupted time series approach, we evaluated pre-period outcome trends between the intervention and comparison group using visual inspection and it was deemed appropriate [50]. For added flexibility, pre-period outcome trends that were not parallel or experienced fluctuation, additional model terms of time and its interaction with the treatment indicator were included to account for these differences. Lastly, post-estimation of these models produced estimated monthly insurance and screening rates over the 36-month study period, with the addition of a post-period counterfactual trend for the intervention group for ease of interpretation. Statistical significance was reported at the *p*-value < 0.05 level. Statistical software R/RStudio version 4.0.5 was used, including packages *geepack* and *emmeans* for statistical modelling and post-estimation procedures.

We estimated the effect of the treatment on the treated to determine whether tool use was associated with gaining and maintaining Medicaid coverage. We summarized the number of patients with a tool instance in Oregon (97% of tool instances were in Oregon) and assessed their baseline and follow-up insurance status.

The Institutional Review Board of the Oregon Health & Science University has reviewed and approved this study.

## Results

Table 1 displays the unadjusted characteristics of patients in the intervention and comparison groups. Patients in the intervention group were less likely to be < 138% under the FPL and less likely to be English speaking than those in the comparison group. The distribution of insurance visits was similar in both intervention and comparison groups, composed primarily of Medicaid insured visits over the 36 months. Intervention clinics had higher preventive ratios for breast and cervical cancer screenings and lower ratios for colorectal cancer screenings than the comparison group.

**Table 1:** Clinic-level characteristics among comparison and intervention groups

	Comparison (*n* = 23 clinics)	Intervention (*n* = 23 clinics)	*p*-value^d^
**State of the clinics, n (%)**			0.419
California	9 (39.1)	5 (21.7)	
Ohio	4 (17.4)	4 (17.4)	
Oregon	10 (43.5)	14 (60.9)	
**Demographic characteristics**^**a**^**, Mean (SD)**			
% Female	66.3 (14.6)	65.2 (7.2)	0.167
% Federal poverty level < 138%	62.3 (25.8)	55.0 (20.7)	<.001
% English speaking	86.9 (15.5)	81.4 (17.5)	<.001
% Non-Hispanic non-White or Hispanic	39.3 (29.1)	40.9 (29.8)	0.461
**Insurance rates (per 100 visits)**^**b**^**, Mean (SD)**			
% Uninsured	9.6 (6.8)	9.1 (10.1)	0.020
% Medicaid	67.5 (13.3)	63.2 (13.6)	0.060
% Other^c^	22.9 (13.2)	27.7 (13.6)	0.003
**Cancer screening preventive ratios**^**c**^**, Mean (SD)**			
% Breast cancer screening	55.2 (16.5)	61.7 (15.1)	<.001
% Cervical cancer screening	61.7 (17.9)	66.0 (12.2)	<.001
% Colorectal cancer screening	49.6 (16.6)	44.3 (13.5)	<.001

Note: ^a^Averaged visit across the 18-month pre-study period

^b^Averaged visit paid by Medicaid, other insurance (private/other public) or self-paid across 36-month study period. Medicare insured visits were excluded

^c^Averaged preventive ratio (%) across 36-month study period

^d^P-values were derived from Chi-square tests and t-tests in order to assess the difference in categorical and continuous characteristics between comparison and intervention groups

### Tool effectiveness on insurance coverage – intent-to-treat

**Fig. 1:**
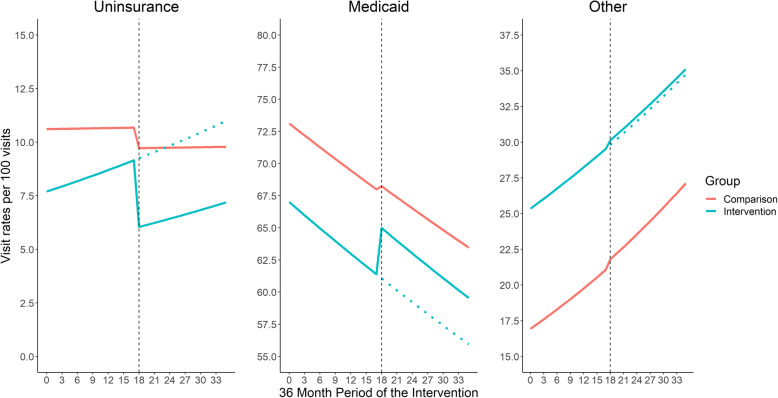
Effectiveness of the enrollment tool on insurance visit rates, 18 months pre- and post-implementation. Note: Other insurance include mainly private insurance, and also other public programs. Dotted black vertical line denotes the implementation of the insurance tool. Clinic-level insurance visit rates were estimated from a Poisson GEE model, adjusted for percent female, < 138% FPL, percent English, percent non-Hispanic White, and state, and utilized an auto-regressive correlation matrix with degree 1. The dotted blue line represents the predicted trend if the enrollment tool had never been implemented

Figure 1 displays the estimated changes in clinic-level rates of uninsured, Medicaid, and other insured visits before and after the tool implementation among the intervention and comparison groups. The intervention group prior to tool implementation had lower uninsured visit rates and Medicaid visit rates than the comparison group.

Immediately following implementation of the enrollment tool, the uninsured visit rate decreased by 21.0% (Adjusted Rate Ratio [RR] = 0.790, 95% CI = 0.621, 1.005, *p* = 0.055) in the intervention group relative to comparison group (Table 2). Though there was an immediate impact on uninsurance rate, the trends in uninsured visit rates (i.e.*,* slope) were similar between intervention and comparison groups (ARR = 1.005; 95% CI = 0,990, 1.021). Similarly, Medicaid-insured visits increased by 4.5% (ARR = 1.045; 95% CI = 1.013, 1.079) immediately after tool implementation in the intervention group compared to the comparison group; the trends in Medicaid visit rates in the intervention and comparison groups were similar (ARR = 1.001; 95% CI = 0.998, 1.004). We observed no immediate impact of the intervention on other insurance type (ARR = 0.999; 95% CI = 0.904, 1.104).

**Table 2:** Insurance rate ratios by intervention periods and clinic group designation

Independent Variables	Uninsured RR (95% CI)	Medicaid RR (95% CI)	Other RR (95% CI)
Time in month	0.999 (0.990–1.009)	**0.996 (0.993–0.998)**	**1.013 (1.004–1.023)**
Intervention period			
Pre-period	Ref	Ref	Ref
Post-period	0.870 (0.736–1.028)	0.908 (0.992–1.037)	1.012 (0.943–1.085)
Clinic group designation			
Comparison	Ref	Ref	Ref
Intervention	0.781 (0.450–1.354)	0.908 (0.791–1.042)	1.530 (0.948–2.470)
Time*Clinic group designation	1.005 (0.990–1.021)	1.001 (0.998–1.004)	0.993 (0.983–1.003)
Intervention period*Clinic group designation	**0.790 (0.621–1.005)**^**a**^	**1.045 (1.013–1.079)**	0.999 (0.904–1.104)
Percent Female	**0.992 (0.988–0.996)**	**1.002 (1.001–1.003)**	0.999 (0.996–1.001)
< 138% FPL	0.998 (0.995–1.002)	1.001 (1.000–1.002)	0.998 (0.995–1.001)
Percent English	**0.981 (0.972–0.990)**	**1.004 (1.002–1.006)**	0.998 (0.991–1.005)
Percent Non-Hispanic White	0.995 (0.989–1.001)	0.999 (0.998–1.001)	**1.004 (1.001–1.008)**
State			
Oregon	Ref	Ref	Ref
California	**0.151 (0.098–0.233)**	1.110 (0.957–1.290)	**1.546 (1.038–2.301)**
Ohio	0.872 (0.510–1.488)	0.919 (0.806–1.048)	**1.410 (1.010–1.969)**

Abbreviations: *RR* Rate Ratios; *Ref* Reference Level; *CI* Confidence Interval

Note: Adjusted rate ratios of insurance were obtained from a multivariate Generalized Estimating Equation Poisson model with robust sandwich variance estimators that account for repeated measures across the study period within each clinic (assuming an auto-regressive correlation structure with degree 1). Bolded text denotes statistical significance (*p*-value < 0.05). ^a^*p* = 0.055

### Tool effectiveness on insurance coverage – effect of the treatment on the treated

Table 3 shows the effect of the treatment on the treated. First, 97% of tool use was observed in Oregon clinics. At the time of tool use, 75% had Medicaid visit, 17% had uninsured visit, and 6% had a privately insured visit. Among those with a tool use, 81% were enrolled in Medicaid 12 months after tool use. Of those, 82% had a Medicaid visit at the time of tool use while 10% had uninsured visits. For the 19% who were never enrolled in Medicaid following tool use, most were uninsured (44%) at the time of tool use. Overall, patients who were uninsured (50%) were the least likely to be enrolled in Medicaid within 12 months of tool use relative to other type of insured patients (Medicaid = 93%, Private = 78%).

**Table 3:** Medicaid enrolment per insurance status at the time of tool use, among patient with one instance of tool use in Oregon

Status at the time of tool use	Total number of patients with tool use	Patient not enrolled in Medicaid 12 months post-tool use; n (%)	Patient enrolled in Medicaid 12 months post-tool use; n (%)	Percent of patient enrolled in Medicaid 12 months post-tool use; (row %)
Total	8651	1690 (19%)	7255, (81%)	8651
Medicaid	6451 (75%)	468 (28%)	5983 (82%)	92.7
Uninsured	1498 (17%)	745 (44%)	753 (10%)	50.3
Other insurance^a^	552 (6%)	120 (27%)	432 (6%)	78.3

Note: Insurance status at the time of tool use based visit date nearest to the tool use to determine baseline insurance status. Medicaid enrollment data were obtained from Oregon Health Authority and linked to EHR data to determine whether patient with tool use were enrolled in Medicaid. Among the 8651 tool instances, 114 patients did not have a subsequent visit in the intervention clinics, 76% of these patients were enrolled in Medicaid. ^a^Other insurance include mainly private insurance, and also other public programs

### Tool effectiveness on Cancer screenings – intent to treat

**Fig. 2:**
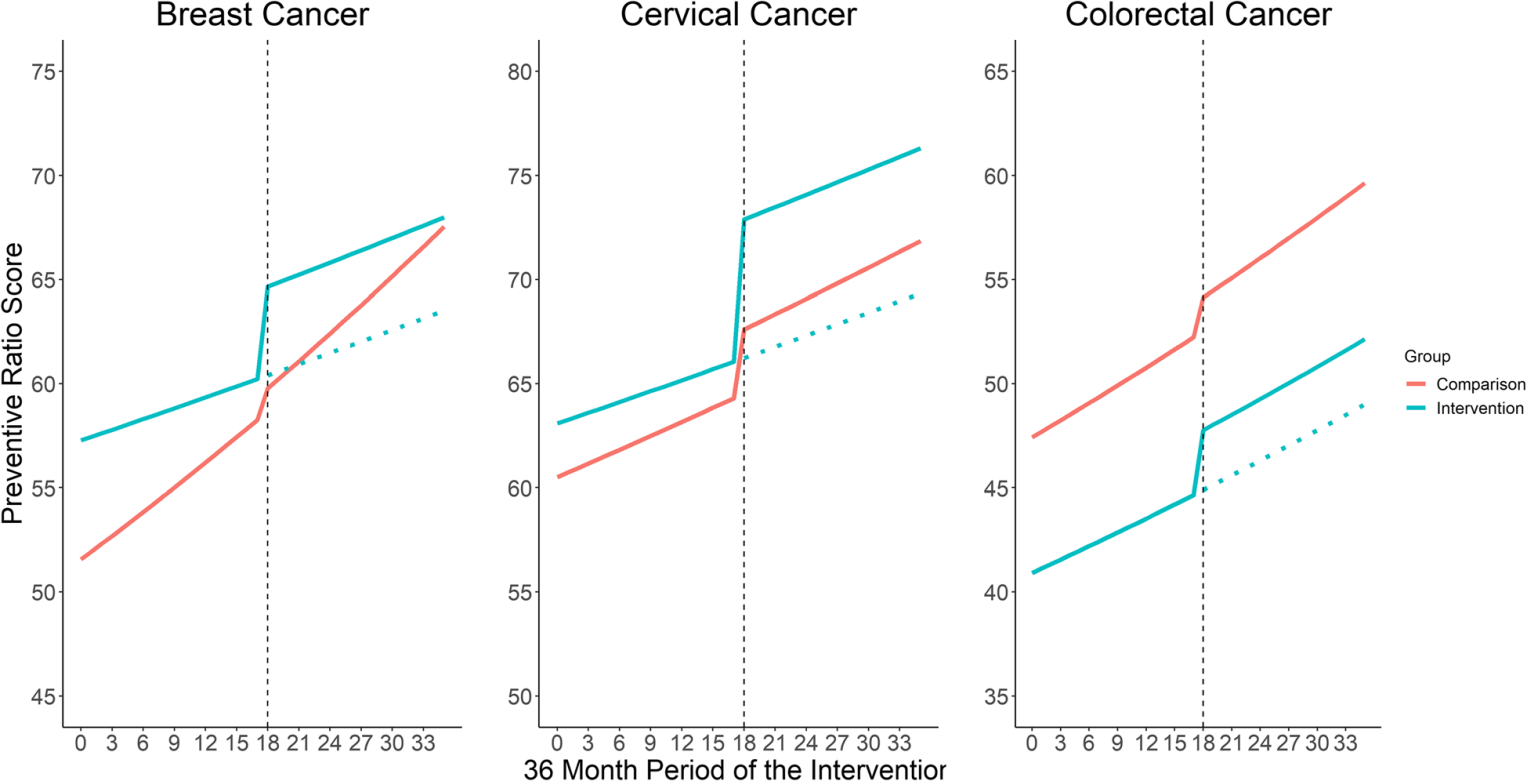
Effectiveness of the enrollemnt tool on cancer screening preventive ratios, 18 months pre- and post-implementation. Note: Preventive ratios represent the percentage of patient-time covered by needed cancer screening. Dotted black vertical line denotes the implementation of the insurance tool. Preventive ratios were estimated from a Poisson GEE model, adjusted for percent female, < 138% FPL, percent English, percent non-Hispanic White, and state, and utilized an auto-regressive correlation matrix with degree 1. The dotted blue line the predicted trend if the enrollment tool had never been implemented

Figure 2 displays the estimated changes in clinic-level screening rates of breast cancer, cervical cancer, and colorectal cancer before and after tool implementation among the intervention and comparison groups. The intervention group, prior to tool implementation, had higher breast cancer and cervical cancer screening preventive ratios and lower colorectal cancer screening preventive ratios than the comparison group. Immediately following implementation of the enrollment tool, cervical screening preventive ratio increased 5.0% (ARR = 1.050, 95% CI = 1.009, 1.093) in the intervention group relative to comparison group (Table 4). Though there was an immediate increase, the trends in cervical cancer preventive ratio were similar between intervention and comparison groups (ARR = 0.999; 95% CI = 0.996, 1.002). For breast and colorectal cancer preventive ratios, the immediate impact and the trends over time were not different between the intervention and comparison groups.

**Table 4:** Rate Ratios of Cancer Screening Preventive Ratios by intervention periods and clinic group designation

Independent Variables	Breast Cancer screening RR (95% CI)	Cervical Cancer screening RR (95% CI)	Colorectal Cancer Screening RR (95% CI)
Time in month	**1.007 (1.003–1.012)**	**1.004 (1.001–1.006)**	**1.006 (1.002–1.009)**
Intervention period			
Pre-period	Ref	Ref	Ref
Post-period	1.019 (0.977–1.062)	**1.048 (1.020–1.076)**	1.031 (0.984–1.080)
Clinic group designation			
Comparison	Ref	Ref	Ref
Intervention	1.111 (0.935–1.319)	1.043 (0.946–1.150)	0.863 (0.701–1.062)
Time*Clinic group designation	0.996 (0.990–1.002)	0.999 (0.996–1.002)	0.999 (0.993–1.006)
Intervention period*Clinic group designation	1.051 (0.995–1.110)	**1.050 (1.009–1.093)**	1.032 (0.971–1.097)
Percent Female	0.999 (0.997–1.002)	1.001 (1.000–1.002)	1.001 (0.998–1.003)
< 138% FPL	1.000 (0.998–1.001)	1.000 (0.999–1.000)	1.001 (0.999–1.002)
Percent English	0.998 (0.995–1.002)	**0.998 (0.996–0.999)**	0.998 (0.995–1.001)
Percent Non-Hispanic White	1.000 (0.998–1.003)	0.999 (0.997–1.000)	1.002 (1.000–1.004)
State			
Oregon	Ref	Ref	Ref
California	1.157 (0.953–1.405)	1.035 (0.911–1.175)	1.194 (0.984–1.448)
Ohio	0.968 (0.784–1.196)	1.083 (0.979–1.197)	1.087 (0.868–1.363)

Abbreviations: *RR* Rate Ratios; *Ref* Reference Level; *CI* Confidence Interval

Note: Adjusted rate ratios of insurance were obtained from a multivariate Generalized Estimating Equation Poisson model with robust sandwich variance estimators that account for repeated measures across the study period within each clinic (assuming an auto-regressive correlation structure with degree 1). Bolded text denotes statistical significance (*p*-value < 0.05)

## Discussion

The health insurance support tool was associated with an initial change in uninsured and Medicaid visits as well as on cancer screenings receipt at the clinic level. Following this initial positive change, the intervention clinics followed similar trends in outcomes relative to the comparison clinics. The tool, however, was only used on a small fraction of patients (16%, see published results on implementation outcomes [41]), which likely explain the small clinic level changes. We believe the low tool adoption rates were due, in part, to the low-intensity of the implementation support, varied uptake of the tool use, and contextual factors (e.g.*,* change in leadership) [41]. First, we did not provide rigid guidance on best practices for implementing this tool but instead let the O&E specialists decide how to use the enrollment tool based on their unique patient populations and needs. As such, the tool was used differently than expected. For instance, O&E specialists in one health system used the tool mainly for outreach, assisting community members who never became established patients at the CHC. Clinics also had varied perspectives of which individuals needed tool use (i.e.*,* who is at risk for uninsurance) and who required support based on resources available through their existing systems of O&E specialists. While it was important for this tool to be adaptable to existing clinic support systems, the absence of these definitions made evaluation of tool impact more challenging. This study shows that EHR-based insurance tool can be useful in clinical practice and may have some associated positive impacts of preventive care, though small with this flexible implementation strategy.

Despite some implementation challenges, O&E specialists did use the tool often for Medicaid re-enrollment, which is essential in avoiding coverage and care gaps. As highlighted by the effect of treatment on the treated analysis, most patients with tool use were enrolled and continued to be enrolled with Medicaid. This result is supported by qualitative interviews with O&E specialists who noted that the enrollment tool was most useful for renewing insurance of Medicaid patients who could be reached through the regional Medicaid program eligibility lists and other forms of in-reach. This finding underscores that EHR-based tools can be developed to assist both clinical and non-clinical personnel in their work. Additionally, it suggests the need for electronic linkage of Medicaid enrollment dates to facilitate clinic efforts in outreaching to patients to assist them in avoiding coverage gaps.

We expected that the tool would be most useful for patients without health insurance. A fraction of tool use instances was associated with an uninsured patient (17%) relative to Medicaid patients (74%). Additionally, most uninsured patients assisted did not gain insurance. This result suggests continued barriers for patients to gain insurance. Previous studies have shown continuous barriers to gaining insurance among various racial and ethnic groups [51, 52]. Continued effort to provide insurance to all is warranted.

### Limitations

This study has some limitations. First, intervention clinics volunteered to be part of the study and are not representative of other CHCs. Second, uninsured patients are less likely to seek care than insured patients and may not be accurately represented in our study. Third, intervention clinics decided on how to use the tool and for whom, rendering effectiveness analysis difficult.

## Conclusion

Insurance support tools integrated within an EHR can be used to streamline health insurance outreach, tracking, and reporting, especially if linked to external sources such as Medicaid enrollment dates for in-reach purposes. Such tools may also have an indirect impact on evidence-based practice interventions, such as cancer screening.

## Data Availability

The datasets used and/or analyzed during the current study are available from the corresponding author on reasonable request.

## Abbreviations

ACA: Affordable Care Act
EHR: Electronic Health Record
CHC: Community Health Center
GEE: Generalized Estimated Eq.
O & E: Outreach and Enrollment
CMS: Centers for Medicare and Medicaid Services
HRSA: Health Resources and Services Administration
HIT: Health Information Technology
PBRN: Practice-Based Research Network
FPL: Federal Poverty Level
