# Correction to: Effectiveness of an insurance enrollment support tool on insurance rates and cancer prevention in community health centers: a quasi-experimental study

**DOI:** 10.1186/s12913-022-07974-8

**Published:** 2022-05-02

**Authors:** Nathalie Huguet, Steele Valenzuela, Miguel Marino, Laura Moreno, Brigit Hatch, Andrea Baron, Deborah J. Cohen, Jennifer E. DeVoe

**Affiliations:** 1 Department of Family Medicine, Oregon Health & Science University, 3181 SW Sam Jackson Park Rd, Portland, OR 97239 USA; 2 Division of Biostatistics, School of Public Health, Oregon Health & Science University – Portland State University, 3181 SW Sam Jackson Park Rd, Portland, OR 97239 USA; 3 Research Department, OCHIN Inc, 1881 SW Naito Pkwy, Portland, OR 97201 USA

**Correction to: BMC Health Serv Res (2021) 21:1186**

**https://doi.org/10.1186/s12913-021-07195-5**

Following publication of the original article [1], the authors identified an error in the author names. The given names and family names of all authors were erroneously transposed.

The incorrect author names are: Huguet Nathalie, Valenzuela Steele, Marino Miguel, Moreno Laura, Hatch Brigit, Baron Andrea, J. Cohen Deborah and E. DeVoe Jennifer

The correct author names are: Nathalie Huguet, Steele Valenzuela, Miguel Marino, Laura Moreno, Brigit Hatch, Andrea Baron, Deborah J. Cohen and Jennifer E. DeVoe

The author group has been updated above and the original article [1] has been corrected.
